# First Cornelia de Lange Syndrome Type 4 Caused by the Gonadal Mosaicism of *RAD21* Deletion

**DOI:** 10.1155/humu/5591607

**Published:** 2026-09-09

**Authors:** Yanchuan Xie, Dongyan Li, Wensheng Li, Yingjie Tao, Li Tang, Liping Li, Guangda Li, Hongwei Jiang

**Affiliations:** ^1^ Central Laboratory, Henan Key Laboratory of Rare Diseases, Endocrinology and Metabolism Center, The First Affiliated Hospital, and College of Clinical Medicine of Henan University of Science and Technology, Luoyang, China, haust.edu.cn; ^2^ Department of Hepatobiliary Surgery, The First Affiliated Hospital, and College of Clinical Medicine of Henan University of Science and Technology, Luoyang, China, haust.edu.cn; ^3^ Institute of Rare Diseases, West China Hospital, Sichuan University, Chengdu, China, scu.edu.cn; ^4^ Henan Key Laboratory of Rare Diseases, Endocrinology and Metabolism Center, The First Affiliated Hospital, and College of Clinical Medicine of Henan University of Science and Technology, Luoyang, China, haust.edu.cn; ^5^ School of Medical Technology and Engineering, Henan University of Science and Technology, Luoyang, China, haust.edu.cn

**Keywords:** Cornelia de Lange syndrome, genotype–phenotype correlation, *RAD21* mutations, whole-genome sequencing

## Abstract

**Background:**

Cornelia de Lange syndrome (CdLS) currently has seven known causative genes, inherited in an autosomal or X‐linked dominant pattern. Clinical features are variable, including dysmorphic facial features, intrauterine and/or postnatal growth retardation, multiple organ system malformations, and neurodevelopmental disorders. Type 4 of CdLS caused by *RAD21* gene has a relatively mild phenotype.

**Methods:**

Here, we report a pair of siblings from a Chinese family who both have similar dysmorphic facial features, cleft palate, spina bifida occulta, and limb phenotypes. Whole‐genome sequencing (WGS) was performed to analyze the genetic basis of their condition. Gap‐PCR was subsequently employed to map the breakpoints in the affected siblings′ peripheral blood samples. Additionally, the copy number status of the parents and the father′s sperm DNA were evaluated using Gap‐PCR and digital PCR (dPCR).

**Results:**

Through WGS analysis, a heterozygous deletion was found in the 8q23.3‐8q24.11 region of both patients, which includes the full‐length *RAD21* gene. And we mapped the breakpoint with the peripheral blood samples of the affected siblings using Gap‐PCR. The parents′ peripheral blood had normal copy number. The father′s sperm DNA was negative for the deletion by both Gap‐PCR and dPCR, whereas the mother′s peripheral blood was also negative, suggesting the deletion is likely due to maternal gonadal mosaicism.

**Conclusions:**

We report a pair of siblings with a complete loss of *RAD21*, with evidence supporting maternal gonadal mosaicism. This information will be helpful for genetic counseling for Type 4 of CdLS.

## 1. Introduction

Cornelia de Lange syndrome is a genetic condition with neurodevelopmental disorders (mild to severe) as the main clinical manifestation, named after the Dutch pediatrician Cornelia de Lange who first reported the syndrome in 1933. The main manifestations are typical facial features, intrauterine and/or postnatal growth retardation, multiple organ system malformations, and neurodevelopmental disorders [1].The incidence of CdLS in newborns is currently believed to be about 1/10,000–1/30,000 [2]. Seven genes have been identified to be related to CdLS—namely, *RAD21*, *SMC1A*, and *SMC3* (structural components of the cohesin complex) and *NIPBL* and *HDAC8* (regulators of the cohesin complex), as well as *BRD4* and *ANKRD11* (nonclassic CdLS) [1, 3, 4]—all of which are dominantly inherited. Notably, *SMC1A* and *HDAC8* follow an X‐linked dominant inheritance pattern. About 70% of cases are related to *NIPBL* gene mutations, that is, Cornelia de Lange syndrome Type 1 (CdLS1, MIM #122470), and the phenotype related to pathogenic mutations of the *RAD21* gene is classified as Cornelia de Lange syndrome Type 4 (CdLS4, MIM #614701). Cognitive defects and physical abnormalities of CdLS4 are milder compared to CdLS1, and *RAD21* mutations are relatively rare, so the proportion of pathogenic mutations of this gene in patients with this syndrome is not very clear at present.

The *RAD21* gene (MIM #606462) is located at 8q24.11, has a total of 14 exons, a full length of 3660 bp, and encodes a 631 amino acid residue RAD21 protein [5]. In eukaryotic cells, the RAD21 protein, as one of the components of the cohesin complexes, provides cohesion for the connection of sister chromatids until the separation of sister chromatids in the late stage of cell division [6]. In addition, RAD21 is also found to be related to DNA damage repair [7]. At present, there are only about 33 cases of CdLS4 patients with clinical information and the molecular evidence reported, of which 11 cases are deletions of chromosome 8q24.1, which include exons 1–14 of *RAD21* and some other genes, and the rest are intragenic pathogenic mutations [3, 4]. There are no reports of *RAD21* mosaicism in these families, but mosaicism has been found in other structural components of the cohesin complex [8]. In this study, we report the clinical characteristics of two siblings caused by *RAD21* gene deletion with possible germline mosaicism due to gonadal mosaicism of the patients′ mother who had no obvious phenotype. To the best of our knowledge, this is the first report documenting gonadal mosaicism in *RAD21*‐related CdLS4. The novelty of our work resides in three key aspects: (i) the identification of a complete heterozygous deletion of the *RAD21* gene in two affected siblings, with no detectable pathogenic variant in either parent′s peripheral blood DNA; (ii) the exclusion of paternal gonadal mosaicism through targeted analysis of the father′s sperm DNA, thereby providing strong evidence that the mutation most likely originates from the maternal germline; and (iii) the provision of direct empirical support for recurrence risk estimation and prenatal genetic counseling in families affected by CdLS4—a contribution not previously reported for pathogenic *RAD2*1 variants.

## 2. Materials and Methods

### 2.1. Samples

We recruited a CdLS pedigree from the Department of Endocrinology, the First Affiliated Hospital of Henan University of Science and Technology, during January 2022 and December 2022. The clinical manifestations of all affected individuals were independently assessed by at least two senior endocrinologists in accordance with the 2018 International Consensus Criteria for Clinical Diagnosis of CdLS [1]. All experimental protocols were approved by the Ethics Committee of the First Affiliated Hospital of Henan University of Science and Technology (Approval No.: K‐2025‐B033). Written informed consent was obtained from all participants or their legal guardians before enrolment. All study procedures were conducted in accordance with the Declaration of Helsinki. Clinical data, peripheral blood, and sperm samples have been collected.

### 2.2. Whole‐Genome Sequencing

HiPure Universal DNA Kit (Magen Biotechnology) was used to extract genomic DNA from 200 ul of blood samples, and the extracted DNA samples were measured using the Qubit 4 Fluorometer (Thermo). The DNA extracted from the patient′s blood was subjected to whole‐genome sequencing using the DNBSEQ‐T7 sequencing platform of BGI. The off‐machine data was aligned with the reference genome sequence (GRCh38) using the BWA software, and the variant information was extracted using the Saile calling algorithm developed based on the GATK Haplotype Caller. The variants were filtered for quality control: read depths > 6, genotype qualities > 20; variants with low mutation frequency in blood (variant allele frequency < 0.01) were selected. The Varseq software was used to annotate the population frequency of the variants (gnomAD database, dbSNP, 1000 Genomes), and variants with MAF (minor allele frequency) ≥0.005 were filtered out; variants annotated as Benign in the ClinVar database were filtered out; variations in the coding region and splicing region were retained. Additionally, copy‐number variants (CNVs) were inferred from WGS data via CNVnator based on read‐depth signals, with a bin size set to 1000 bp. Prior to CNV partitioning and calling, read‐depth histograms were generated, normalized, and corrected for GC bias following the built‐in standard procedures of CNVnator.

### 2.3. Gap‐PCR Detection

Gap‐PCR was used to detect the copy number variations found in the aforementioned sequencing. The method of DNA extraction was as described previously, using the HiPure Universal DNA Kit for sperm DNA purification. Primer Express Software (Applied Biosystems) was used to design primers to amplify the deletion of the *RAD*21 gene. Primer sequences were designed based on the detection site: chr8:116612942–117090248 Het Deletion (GRCh38), with the sequences *RAD*21‐F: GTCAGACCTTTCTCCAGGCACT, and *RAD*21‐R: CAACAAAGACAGGCTCCTTACACAG.

### 2.4. Digital PCR (dPCR) Validation

To rule out low‐level mosaicism in the paternal germline, dPCR was performed on the father′s sperm DNA. The assay was conducted on the DQ24 Digital PCR System (Berry Genomics, Beijing, China), which generates approximately 23,000 partitions per 20 *μ*L reaction. The limit of detection (LoD) was estimated at 10^−4^–10^−3^. For the sperm sample, five parallel replicate reactions were set up, along with two negative controls. The dPCR reaction mixture (20 *μ*L total volume) comprised 5 *μ*L of 4 × dPCR Probe Master Mix (Cy5.5), 1 *μ*L each of forward and reverse primers for the target gene *RAD*21 (10 *μ*M), 0.5 *μ*L of *RAD*21 probe (10 *μ*M), 1 *μ*L each of forward and reverse primers for the reference gene *RPP30* (10 *μ*M), 0.5 *μ*L of *RPP*30 probe (10 *μ*M), template DNA (4 *μ*L), and made up to 20 *μ*L with nuclease‐free water. Primer and probe sequences were designed based on the deleted region chr8:116610001–117090000 (GRCh38): *RAD*21‐F TTTTGGAATGGATGATCGTGAGAT, *RAD*21‐R TCTCATTCAGATTGCTGGTGCT, and *RAD*21‐P FAM‐TGAGAGAAGGCAGTGCT‐MGB; for *RPP*30, the primers were AGATTTGGACCTGCGAGCG (F) and GAGCGGCTGTCTCCACAAGT (R), and the probe was HEX‐TTCTGACCTGAAGGCTCTGCGCG‐BHQ. The amplification protocol consisted of an initial incubation at 60°C for 300 s and 95°C for 300 s, followed by 45 cycles of 95°C for 20 s and 60°C for 45 s. Data were analyzed in 1D Amplitude mode, with fluorescence thresholds set for the FAM (*RAD*21) and HEX (*RPP*30) channels to distinguish positive from negative droplets. The deletion ratio was calculated as (*RPP*30 copy number − *R* *A* *D*21 copy number)/*RPP*30 copy number, and the deletion status was determined by comparing this ratio with that of the negative control and in combination with the sensitivity.

## 3. Results

### 3.1. Case Information

Patient 1 is a 9.3‐year‐old boy born to G1P1, 24 years old mom via vaginal delivery at full term. The parents are nonconsanguineous Chinese Han parents. There is no family history of congenital diseases. Prenatal examinations were normal, fetal growth was normal, no intrauterine growth restriction was found, and the pregnancy was uneventful. At birth, the head circumference was 33 cm (10th percentile). Growth has been normal since then. Currently, his height is 138 cm (50th percentile), and his weight is 31.7 kg (50th percentile).

**Figure 1:**
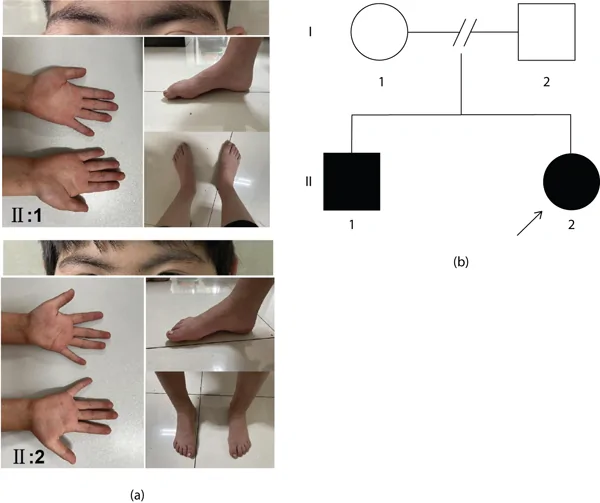
Patient Phenotypic Features and Sequencing Validation. (a) Patient 1 (II: 1) and Patient 2 (II: 2) both exhibit synophrys and thick eyebrows, as well as small hands and feet. (b) Pedigree of the family with a heterozygous deletion found in the 8q23.3‐8q24.11 region.

The patient′s facial features include synophrys, thick eyebrows, and a depressed nasal bridge. The patient also has some degree of skeletal abnormalities (Figure 1a), including small hands and feet. X‐ray revealed spina bifida occulta at the first sacral vertebra (S1). The patient′s congenital cleft palate was successfully repaired. He had normal vision and hearing. He was evaluated at 9 years old and found to have normal intelligence and motor development. Based on the 2018 CdLS International Consensus Clinical Features [1], these siblings were scored 5 points.

Patient 2 is a female and the second child of the couple (Figure 1b). Prenatal examinations showed normal fetal development, no intrauterine growth restriction, and the pregnancy was uneventful. The birth weight, length, and head circumference were all in a normal range. She is 6.6 years old, with a height of 95 cm (< 3rd percentile) and a weight of 20.3 kg (25th–50th percentile).

Patient 2 shares similar dysmorphic features with her brother, including synophrys, thick eyebrows, a depressed nasal bridge, and small hands and feet; x‐ray examination confirmed spina bifida occulta at S1, as in her brother. Additionally, the patient has a short stature and delayed bone age (Figure 1a). The cleft palate was also surgically repaired. No significant abnormalities were found in vision or hearing. She has normal cognitive development. An electrocardiogram showed sinus rhythmia, and no cardiac structural abnormalities were found. Based on the 2018 CdLS International Consensus Clinical Features [1], these siblings were scored 6 points.

### 3.2. Whole‐Genome Sequencing and Mutation Validation

The clinical features are suggestive CdLS. Whole‐genome sequencing was performed on peripheral blood DNA from both patients (Patient 1 and Patient 2). A 0.48 Mb heterozygous deletion at 8q23.3‐24.11 (chr8:116610001–117090000) was identified in both siblings. According to the ACMG/ClinGen CNV pathogenicity rating criteria, this CNV was classified as pathogenic, with an overall score of 1.0. We further performed Gap‐PCR on peripheral blood samples from the two patients as well as their parents to validate this deletion and determine its parental origin (see upcoming discussion).

**Figure 2:**
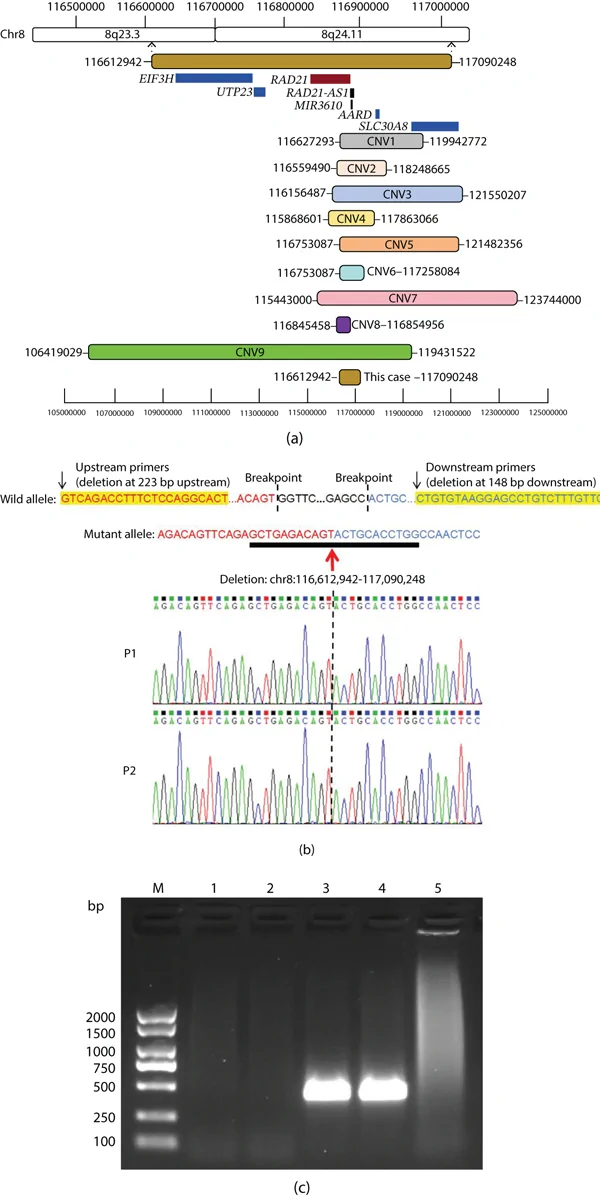
Gene pattern diagram and gap‐PCR verification. (a) Genome microdeletion region (yellow rectangle), including the full length of *RAD21* (red rectangle). The deletion involves five protein‐coding genes (*EIF3H*, *UTP23*, *RAD21*, *AARD*, and *SLC30A8*) and two noncoding genes (*RAD21-AS1* and *MIR3610*) (protein‐coding genes indicated by blue rectangles and nonprotein‐coding by black rectangles). The previously reported CNV deletion region (numbered CNV1‐9) is shown below with rectangles of different colors. (b) Gap‐PCR verification showed that the patient has a deletion at chr8:116612942‐117090248 (red arrow), and the breakpoint was indicated by a dotted line. Not all the bases deleted within the breakpoint were shown. The base sequences framed in yellow are the positions of the upstream and downstream primers. (c) Gap‐PCR products from this family member were run on agarose gel electrophoresis. The sources of the products in Lanes 1–5 are paternal peripheral blood (Lane 1), maternal peripheral blood (Lane 2), Patient 1 peripheral blood (Lane 3), Patient 2 peripheral blood (Lane 4), and paternal semen (Lane 5). Marker molecule on the far left.

A pair of primers was designed based on WGS for Gap‐PCR, which confirmed a deletion at chr8:116612942–117090248 (Figure 2b). The results showed that the affected brother (Lane 3) and younger sister (Lane 4) both displayed a 409 bp band, whereas neither the father (Lane 1) nor the mother (Lane 2) had this band (Figure 2c). Therefore, the deletion at 8q23.3‐24.11 in this family is not present in the parents′ blood samples.

This region encompasses seven genes in total: five protein‐coding genes (*EIF3H*, *UTP23*, *RAD*21, *AARD*, and *SLC30A8*) and two noncoding genes (*RAD*21*-AS1* and *MIR3610*). Of these genes, only *RAD*21 is a known haploinsufficient gene linked to CdLS4. In contrast, the remaining four protein‐coding genes (*EIF3H*, *UTP*23, *AARD*, and *SLC30A8*) have no established disease associations, including with CdLS4, according to the OMIM and ClinGen databases. Additionally, no evidence of haploinsufficiency has been documented for these genes. Furthermore, no pathogenic variants of the four genes have been reported in ClinVar and gnomAD.

### 3.3. Assessment of Mosaicism in Germline/Somatic Cells

As two children shared the same clinical features and genotypes, we suspect gonadal mosaicism. We first examine father′s sperm. We used the father′s sperm DNA samples for Gap‐PCR to further confirm whether the sperm contained the deletion at 8q23.3‐24.11. Gel electrophoresis shows that no detectable 409 bp fragment (Figure 2c, Lane 5); this result is consistent with our dPCR of the sperm (data not shown), suggesting the gonadal mosaicism is from mother. However, we were not able to confirm experimentally as mother′s gonadal tissue was unavailable for testing.

### 3.4. Literature Review

We have compiled the clinical data of 33 reported cases of *RAD21* variant patients, including the two patients in this study (clinical information, see Table S1). According to statistics, male patients account for 54%, and female patients account for 46%, with a ratio of 1.2:1, which is close to the overall reported male‐to‐female ratio of CdLs cases (1.4:1) [9, 10]. Currently, most reported cases of *RAD21* are in the America and Europe, with few reports in the East Asian population. The most common clinical phenotypes in individuals affected by *RAD21* mutations include microcephaly (54.5%), thick eyebrows (51.5%), synophrys (39.4%), long eyelashes (36.4%), short nose (27.3%), broad nasal bridge (27.3%), cleft palate (33.3%), and abnormalities in the development of the fifth finger (30.3%), and long and/or flat philtrum (36.4%). These craniofacial features are consistent with the facial expressions of other types of CdLS. The facial features in our patients are consistent with other cases, but small hands and feet (12.1%) are relatively rare. The limb abnormalities reported in CdLS4 cases mainly include transverse palm (15.2%), abnormalities in the development of the fifth finger (30.3%), and limited elbow movement (15.2%), which were not found in our two patients. In addition, the nervous system of individuals affected by *RAD21* is mainly language delay and slow motor development (54.5%), which is very variable [11]. It has been reported that the proportion of high palate arch and cleft palate in patients with *SMC1A* is higher than in other types of CdLS [12], which is also more common in the *RAD21-*related patients (33.3%). A third of CdLS patients can have heart defects, such as patent ductus arteriosus (PDA) and ventricular septal defect (VSD) [8,9], which is less common in *RAD21* patients (15.2%) and not present in our patients. Other abnormalities such as exostoses, pectus carinatum/pectus excavatum, and holoprosencephaly were absent in both reported CdLS4 cases and our patients; however, spina bifida occulta was present in our two cases.

## 4. Discussion

The RAD21 protein is one of the components of the cohesin protein complex. The dimer of SMC1A and SMC3 forms a hinge structure, connected to STAG1/2 and RAD21, which play a central structural role, thus forming a “ring.” This ring‐shaped protein polymer also has some regulatory proteins, such as NIPBL and HDAC8 [13]. The cohesin protein complex can maintain chromatin structure during cell division and transcription regulation [14], and variations in its subunits or regulatory factors can all lead to CdLS [15].

To further elucidate the genotype–phenotype correlation in CdLS4 patients harboring *RAD21* deletions, we compared the clinical features of our two patients with those of nine previously reported cases with *RAD21*‐associated CNVs (CNV1‐CNV9, summarized in Table S1 and Figure 2a). Common phenotypic features among these nine cases included microcephaly (5/9, 55.6%), thick eyebrows (5/9, 55.6%), synophrys (4/9, 44.4%), long eyelashes (3/9, 33.3%), cleft palate (3/9, 33.3%), and abnormal morphology of the fifth finger (3/9, 33.3%). Cardiac abnormalities were documented in two cases (2/9, 22.2%), and multiple exostoses in four cases (4/9, 44.4%). In particular, both probands in this study presented with spina bifida occulta, a vertebral anomaly not previously documented in *RAD21* deletion cases, thereby expanding the known clinical spectrum of CdLS4.

Among these CNV cases, Lei et al. [16] described a male patient with a deletion spanning 8q23.3‐q24.13; notably, this individual and our two probands are of Chinese Han ethnicity. Shared clinical features across our probands and the case reported by Lei et al. [16] include thick eyebrows, a depressed nasal bridge, cleft palate, and small hands and feet. With regard to trunk skeletal abnormalities, both of our probands exhibited spina bifida occulta, whereas the patient described by Lei et al. [16] presented with scoliosis–suggesting that *RAD21* haploinsufficiency may confer variable expressivity of vertebral anomalies. Especially, the patient reported by Lei et al. [16] displayed a more severe phenotype, including congenital heart defects and intellectual disability—features absent in our two siblings. This phenotypic disparity may be attributable to the larger deletion size in the Lei et al. [16] case, which encompassed additional pathogenic genes, such as EXT1—a gene implicated in Langer–Giedion syndrome—and known to cause exostoses and other skeletal abnormalities. In contrast, aside from *RAD21*, the other protein‐coding genes located within the deleted region in our patients (*EIF3H*, *UTP23*, *AARD*, and *SLC30A8*)—as noted above—currently lack documented evidence of pathogenicity or dosage sensitivity. Therefore, the milder phenotype observed in our two patients is consistent with isolated a pure *RAD21* haploinsufficiency—uncomplicated by dosage‐sensitive genes located outside the *RAD21* locus. Importantly, this genetic configuration allows us to attribute the spina bifida occulta observed in both probands specifically to *RAD21* haploinsufficiency, rather than contributing genes such as *EXT1*, which is located outside our deleted interval and has been associated with skeletal abnormalities. Thus, this vertebral anomaly can be recognized as a genuine novel phenotype within the CdLS4 spectrum.

Moreover, the absence of cardiac malformations, exostoses, and microcephaly in our patients—coupled with their preserved cognitive function—reinforces the concept that *RAD21* deletions can produce a relatively mild clinical presentation when the deleted interval is confined to *RAD21* alone and does not encompass other disease‐associated genes. These findings emphasize the critical importance of high‐resolution breakpoint mapping to enable precise genotype‐phenotype correlations and informed genetic counseling.

Importantly, conventional Gap‐PCR has limited sensitivity for detecting low‐level mosaicism, with a typical detection threshold of approximately 5%–10%. To rule out false‐negative results, we employed dPCR for validation; this method has a detection limit as low as 10^−4^–10^−3^ [17], which is significantly higher than that of conventional PCR. The dPCR results were also negative. Although we cannot rule out extremely low‐level mosaicism below the dPCR detection threshold, the combined data strongly suggest the deletion is undetectable in the father′s sperm, further supporting a maternal origin of gonadal mosaicism. We also propose that single sperm sequencing may be adopted in future studies to achieve higher detection sensitivity.

It should be noted that a negative Gap‐PCR result for the mother′s peripheral blood merely indicates that the deletion fraction in her blood cells falls below the assay′s detection limit (5%–10%). This finding does not completely exclude the presence of low‐level somatic mosaicism. If the deletion is restricted solely to ovarian tissue and undetectable in peripheral blood, the case represents pure gonadal mosaicism. In contrast, if the deletion exists at extremely low levels in both gonads and other somatic tissues, it is defined as germline‐somatic mosaicism. Because ovarian and oocyte samples were not available from the mother, we are currently unable to differentiate between these two conditions. Further investigations using high‐depth targeted sequencing (e.g., dPCR) on her peripheral blood DNA or other readily accessible specimens, such as buccal swabs and urinary sediment, may help verify low‐level somatic mosaicism. Such analyses are critical for precise recurrence risk assessment and the delivery of appropriate genetic counseling for this family.

Gonadal mosaicism is a relatively rare but important cause of recurrence of familial genetic diseases [18] and critical for genetic counseling. Gonadal mosaicism was previously reported in the *NIPBL*‐related CdLS. In the present family, the occurrence of two affected siblings carrying the same *RAD21* deletion, along with negative Gap‐PCR results in both parents′ peripheral blood and the father′s sperm, strongly supports maternal gonadal mosaicism. Quantifying the recurrence risk for genetic counseling is of particular importance in such circumstances. The International CdLS Consensus Group, based on their joint experience in 560 families with at least one individual carrying a causal *NIPBL* variant, reported that the familial recurrence risk attributable to gonadal mosaicism is 0.89% [1]. Given that *RAD21*, like *NIPBL*, is an autosomal gene and not X‐linked, this empirical estimate is likely applicable to the present family. The consensus statement also emphasized that genetic counseling for families with *RAD21* variants remains particularly challenging due to the remarkable phenotypic variability observed even within the same family. Therefore, we recommend that this family be offered prenatal genetic testing or preimplantation genetic diagnosis (PGD) in future pregnancies, with the understanding that the actual recurrence risk depends on the proportion of affected gametes in the maternal germline—a factor that could be further elucidated if maternal germ cells become available for testing—which would confirm the gonadal mosaicism of the *RAD21* mutation.

## 5. Conclusions

In summary, we have identified a new CNV variation chr8:116612942–117090248 deletion in a pair of siblings in a Han Chinese family, which includes the whole‐gene deletion of *RAD21*, leading to CdLS Type 4, and verified the mosaic level of the father′s sperm cells. We have summarized the clinical information and mutation information of *RAD21*‐related CdLS cases, which will help in the future for correct genetic counseling and appropriate follow‐up for patients with CdLS Type 4.

## Author Contributions

L.L. and W.L. collected the clinical data of the patients. Y.X., L.T., and Y.T. were responsible for the analysis of the experimental data. Y.X. and D.L. were responsible for the experimental design and wrote the first draft of the manuscript. G.L. and H.J. reviewed and edited the manuscript.

## Funding

This study was supported by the Medical Science and Technology Research and Development Plan Major Project Jointly Constructed by the Henan Province and Ministerial Departments in China (No.SBGJ202301010) and 1.3.5 Project for Disciplines of Excellence by West China Hospital of Sichuan University (ZYJC20002).

## Disclosure

We confirm that the work is original and has not been under review or published elsewhere. All authors have read and agreed to the published version of the manuscript.

## Ethics Statement

All experimental protocols were approved by the Ethics Committee of the First Affiliated Hospital of Henan University of Science and Technology (Approval No.: K‐2025‐B033). Written informed consent was obtained from all participants or their legal guardians before enrolment. All study procedures were conducted in accordance with the Declaration of Helsinki. To protect patient privacy, facial images presented in this study have been deidentified. All original identifiable information (including but not limited to imaging data, clinical history, and laboratory test results) is accessible only to the core research team members responsible for data collection and analysis, stored in an encrypted institutional database, and is not disclosed to any external parties.

## Conflicts of Interest

The authors declare no conflicts of interest.

## Supporting information


**Supporting Information** Additional supporting information can be found online in the Supporting Information section. Table S1: Summary of reported clinical characteristics of patients with Cornelia de Lange syndrome Type 4. The table summarizes the clinical data of 33 reported cases of *RAD21* variant patients, including the two patients in this study.

## Data Availability

The data that support the findings of this study are available from the corresponding author upon reasonable request.

## References

[1] Kline A. D. , Moss J. F. , Selicorni A. , Bisgaard A. M. , Deardorff M. A. , Gillett P. M. , Ishman S. L. , Kerr L. M. , Levin A. V. , Mulder P. A. , Ramos F. J. , Wierzba J. , Ajmone P. F. , Axtell D. , Blagowidow N. , Cereda A. , Costantino A. , Cormier-Daire V. , FitzPatrick D. , Grados M. , Groves L. , Guthrie W. , Huisman S. , Kaiser F. J. , Koekkoek G. , Levis M. , Mariani M. , McCleery J. P. , Menke L. A. , Metrena A. , O’Connor J. , Oliver C. , Pie J. , Piening S. , Potter C. J. , Quaglio A. L. , Redeker E. , Richman D. , Rigamonti C. , Shi A. , Tümer Z. , van Balkom I. D. C. , and Hennekam R. C. , Diagnosis and Management of Cornelia De Lange Syndrome: First International Consensus Statement, Nature Reviews Genetics. (2018) 19, no. 10, 649–666, 10.1038/s41576-018-0031-0, 29995837. PMC7136165 29995837

[2] Bottai D. , Spreafico M. , Pistocchi A. , Fazio G. , Adami R. , Grazioli P. , Canu A. , Bragato C. , Rigamonti S. , Parodi C. , Cazzaniga G. , Biondi A. , Cotelli F. , Selicorni A. , and Massa V. , Modeling Cornelia De Lange Syndrome In Vitro and In Vivo Reveals a Role for Cohesin Complex in Neuronal Survival and Differentiation, Human Molecular Genetics. (2019) 28, no. 1, 64–73, 10.1093/hmg/ddy329, 30239720. 30239720

[3] Abarca-Barriga H. H. , Punil Luciano R. , and Vásquez S. F. , Cornelia De Lange Syndrome Caused by an Intragenic Heterozygous Deletion in RAD21 Detected Through Very-High-Resolution Chromosomal Microarray Analysis, Genes. (2023) 14, no. 12, 10.3390/genes14122212, 38137034. PMC10742884 38137034

[4] De Falco A. , De Brasi D. , Della Monica M. , Cesario C. , Petrocchi S. , Novelli A. , D’Alterio S. , Iolascon A. , Capasso M. , and Piscopo C. , A Novel Variant in RAD21 in Cornelia De Lange Syndrome Type 4: Case Report and Bioinformatic Analysis, Genes. (2023) 14, no. 1, 10.3390/genes14010119, 36672860. PMC9859063 36672860

[5] McKay M. J. , Troelstra C. , Kanaar R. , Smit B. , Hagemeijer A. , Bootsma D. , and Hoeijmakers J. H. J. , Sequence Conservation of therad21 Schizosaccharomyces pombeDNA Double-Strand Break Repair Gene in Human and Mouse, Genomics. (1996) 36, no. 2, 305–315, 10.1006/geno.1996.0466, 8812457. 8812457

[6] Sumara I. , Vorlaufer E. , Gieffers C. , Peters B. H. , and Peters J. M. , Characterization of Vertebrate Cohesin Complexes and Their Regulation in Prophase, Journal of Cell Biology. (2000) 151, no. 4, 749–762, 10.1083/jcb.151.4.749, 11076961. 11076961 PMC2169443

[7] Xu H. , Balakrishnan K. , Malaterre J. , Beasley M. , Yan Y. , Essers J. , Appeldoorn E. , Thomaszewski J. M. , Vazquez M. , Verschoor S. , Lavin M. F. , Bertonchello I. , Ramsay R. G. , and McKay M. J. , Rad21-Cohesin Haploinsufficiency Impedes DNA Repair and Enhances Gastrointestinal Radiosensitivity in Mice, PLoS One. (2010) 5, no. 8, e12112, 10.1371/journal.pone.0012112, 20711430. 20711430 PMC2920816

[8] Ansari M. , Poke G. , Ferry Q. , Williamson K. , Aldridge R. , Meynert A. M. , Bengani H. , Chan C. Y. , Kayserili H. , Avci Ş. , Hennekam R. C. M. , Lampe A. K. , Redeker E. , Homfray T. , Ross A. , Falkenberg Smeland M. , Mansour S. , Parker M. J. , Cook J. A. , Splitt M. , Fisher R. B. , Fryer A. , Magee A. C. , Wilkie A. , Barnicoat A. , Brady A. F. , Cooper N. S. , Mercer C. , Deshpande C. , Bennett C. P. , Pilz D. T. , Ruddy D. , Cilliers D. , Johnson D. S. , Josifova D. , Rosser E. , Thompson E. M. , Wakeling E. , Kinning E. , Stewart F. , Flinter F. , Girisha K. M. , Cox H. , Firth H. V. , Kingston H. , Wee J. S. , Hurst J. A. , Clayton-Smith J. , Tolmie J. , Vogt J. , Tatton–Brown K. , Chandler K. , Prescott K. , Wilson L. , Behnam M. , McEntagart M. , Davidson R. , Lynch S. A. , Sisodiya S. , Mehta S. G. , McKee S. A. , Mohammed S. , Holden S. , Park S. M. , Holder S. E. , Harrison V. , McConnell V. , Lam W. K. , Green A. J. , Donnai D. , Bitner-Glindzicz M. , Donnelly D. E. , Nellåker C. , Taylor M. S. , and FitzPatrick D. R. , Genetic Heterogeneity in Cornelia De Lange Syndrome (CdLS) and CdLS-Like Phenotypes With Observed and Predicted Levels of Mosaicism, Journal of Medical Genetics. (2014) 51, no. 10, 659–668, 10.1136/jmedgenet-2014-102573, 25125236. 25125236 PMC4173748

[9] Hei M. , Gao X. , and Wu L. , Clinical and Genetic Study of 20 Patients From China With Cornelia De Lange Syndrome, BMC Pediatrics. (2018) 18, no. 1, 10.1186/s12887-018-1004-3, 29452578. PMC5815176 29452578

[10] Kline A. D. , Grados M. , Sponseller P. , Levy H. P. , Blagowidow N. , Schoedel C. , Rampolla J. , Clemens D. K. , Krantz I. , Kimball A. , Pichard C. , and Tuchman D. , Natural History of Aging in Cornelia De Lange Syndrome, American Journal of Medical Genetics Part C, Seminars In Medical Genetics. (2007) 145, no. 3, 248–260, 10.1002/ajmg.c.30137. PMC4902018 17640042

[11] Deardorff M. A. , Wilde J. J. , Albrecht M. , Dickinson E. , Tennstedt S. , Braunholz D. , Mönnich M. , Yan Y. , Xu W. , Gil-Rodríguez M. C. , Clark D. , Hakonarson H. , Halbach S. , Michelis L. D. , Rampuria A. , Rossier E. , Spranger S. , van Maldergem L. , Lynch S. A. , Gillessen-Kaesbach G. , Lüdecke H. J. , Ramsay R. G. , McKay M. J. , Krantz I. D. , Xu H. , Horsfield J. A. , and Kaiser F. J. , RAD21 Mutations Cause a Human Cohesinopathy, American Journal Of Human Genetics. (2012) 90, no. 6, 1014–1027, 10.1016/j.ajhg.2012.04.019, 22633399. 22633399 PMC3370273

[12] Parenti I. , Rovina D. , Masciadri M. , Cereda A. , Azzollini J. , Picinelli C. , Limongelli G. , Finelli P. , Selicorni A. , Russo S. , and Gervasini C. , Overall and Allele-Specific Expression of the SMC1A Gene in Female Cornelia De Lange Syndrome Patients and Healthy Controls, Epigenetics. (2014) 9, no. 7, 973–979. 24756084 10.4161/epi.28903 PMC4143412

[13] Yuan B. , Neira J. , Pehlivan D. , Santiago-Sim T. , Song X. , Rosenfeld J. , Posey J. E. , Patel V. , Jin W. , Adam M. P. , Baple E. L. , Dean J. , Fong C. T. , Hickey S. E. , Hudgins L. , Leon E. , Madan-Khetarpal S. , Rawlins L. , Rustad C. F. , Stray-Pedersen A. , Tveten K. , Wenger O. , Diaz J. , Jenkins L. , Martin L. , McGuire M. , Pietryga M. , Ramsdell L. , Slattery L. , DDD Study , Abid F. , Bertuch A. A. , Grange D. , Immken L. , Schaaf C. P. , van Esch H. , Bi W. , Cheung S. W. , Breman A. M. , Smith J. L. , Shaw C. , Crosby A. H. , Eng C. , Yang Y. , Lupski J. R. , Xiao R. , and Liu P. , Clinical Exome Sequencing Reveals Locus Heterogeneity and Phenotypic Variability of Cohesinopathies, Genetics in Medicine. (2019) 21, no. 3, 663–675, 10.1038/s41436-018-0085-6, 30158690. 30158690 PMC6395558

[14] Zhang Y. and Fang Y. D. , Progresses on the Structure and Function of Cohesin, Yi Chuan. (2020) 42, no. 1, 57–72, 10.16288/j.yczz.19-288, 31956097. 31956097

[15] Parenti I. and Kaiser F. J. , Cornelia de Lange Syndrome as Paradigm of Chromatinopathies, Frontiers In Neuroscience. (2021) 15, 774950, 10.3389/fnins.2021.774950, 34803598. 34803598 PMC8603810

[16] Lei M. , Liang D. , Yang Y. , Mitsuhashi S. , Katoh K. , Miyake N. , Frith M. C. , Wu L. , and Matsumoto N. , Long-Read DNA Sequencing Fully Characterized Chromothripsis in a Patient With Langer-Giedion Syndrome and Cornelia De Lange Syndrome-4, Journal of Human Genetics. (2020) 65, no. 8, 667–674, 10.1038/s10038-020-0754-6, 32296131. 32296131 PMC7324355

[17] Li X. , Huang S. , Wang G. , Kang D. , Han M. , Wu X. , Yang J. , Zheng Q. , Zhao C. , Yuan Y. , and Dai P. , Quantitative Assessment of Low-Level Parental Mosaicism of SNVs and CNVs in Waardenburg Syndrome, Human Genetics. (2023) 142, no. 3, 419–430, 10.1007/s00439-022-02517-x, 36576601. 36576601

[18] Drost J. B. and Lee W. R. , The Developmental Basis for Germline Mosaicism in Mouse and Drosophila Melanogaster, Genetica. (1998) 102, 421–443, 10.1023/A:1017002221520, 9720293. 9720293

